# Supplementary material for: Discovery and pharmacophoric characterization of chemokine network inhibitors using phage-display, saturation mutagenesis and computational modelling
Source: Nat Commun. 2023 Sep 16;14:5763. doi: 10.1038/s41467-023-41488-z (PMC10505172; doi:10.1038/s41467-023-41488-z)
Supplement: Supplementary file 4 — Source Data [file 41467_2023_41488_MOESM4_ESM.zip › SourceData/scripts/xNNKs_20230808183658.tar!/xNNKs/analysis/heatmaps/SV_HD2X_methionine_heatmap.pdf]

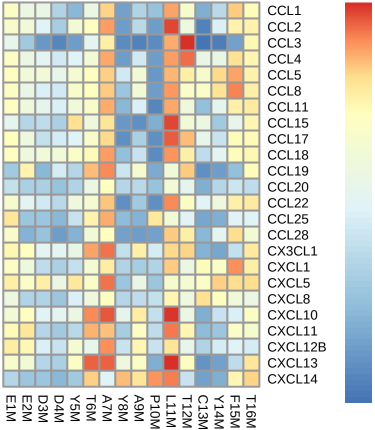

CCL1
CCL2
CCL3
CCL4
CCL5
CCL8
CCL11
CCL15
CCL17
CCL18
CCL19
CCL20
CCL22
CCL25
CCL28
CX3CL1
CXCL1
CXCL5
CXCL8
CXCL10
CXCL11
CXCL12B
CXCL13
CXCL14
E1M
E2M
D3M
D4M
Y5M
T6M
A7M
Y8M
A9M
P10M
L11M
T12M
C13M
Y14M
F15M
T16M
2
0
−2
−4
−6
−8
